# Problematic Internet Use among University Students and Its Relationship with Social Skills

**DOI:** 10.3390/brainsci11101301

**Published:** 2021-09-30

**Authors:** Miriam Romero-López, Carmen Pichardo, Isabel De Hoces, Trinidad García-Berbén

**Affiliations:** Department of Evolutive and Educational Psychology, University of Granada, 18071 Granada, Spain; miriam@ugr.es (M.R.-L.); isadehoces@gmail.com (I.D.H.); tgarciab@ugr.es (T.G.-B.)

**Keywords:** internet addiction, age, social skills, empathy, incidence

## Abstract

Internet use has been steadily and unstoppably gaining ground in all areas of life, from recreational activities to the establishment of social relations. However, addictive use of the Internet is a problem that seriously affects some people. Factors that may influence the occurrence of inappropriate internet use include age and social skills. For this reason, the aim of this study is to analyze the influence of social skills and age on the development of problematic internet use in university students. The study involved 514 students enrolled at a university in Spain, who filled in two questionnaires, one on problematic internet use and the other on social skills. Multivariate multiple linear regression models revealed that some social skills variables (conversation and social ease, empathic and positive feeling skills, risk coping) predicted problematic internet use. In addition, age played a role in preference for online social interaction and deficient self-regulation. Younger students were more at risk of having obsessive thoughts related to internet use and of engaging in compulsive internet use compared to older students.

## 1. Introduction

At the end of the 1990s, authors such as Young [1] and Goldberg [2] suggested for the first time that the Internet can generate addictions. Problematic internet use is defined as a person’s inability to control his or her own internet use leading to disruption and impairment in the fulfilment of social, work and personal commitments [3].

Davis [4] and the taxonomy proposed by Montang et al. [5] differentiate between generalized and specific problematic Internet use disorders. Specific disorders include those who are dependent on a specific function of the Internet, such as excessive use of social networking sites or online gambling. Pervasive disorders, on the other hand, involve a general, multidimensional overuse of the Internet and include behaviors such as wasting time online without a clear purpose. Therefore, there is no abuse of specific content but rather an abuse of several channels with different content [4,5].

Studies on this topic have found that one of the most worrying variables and determinants of problematic internet use is social skills [6,7,8]. At present, there is no consensus on the definition of social skills due to the fact that the term social skills is dependent on context, situation, age, gender, familiarity or environment, not being a generalized and unitary trait [9]. However, most definitions state that social skills are those learned social skills that are necessary to relate and interact with other people in an effective and satisfactory way, allowing the individual to successfully resolve social situations [9,10].

In relation to social skills there are two differentiated positions, depending on whether there is problematic use or healthy or appropriate use of the Internet (understood as the use of this tool for a specific purpose and for a reasonable period of time, without any behavioral or cognitive alteration) [4]. On the one hand, some authors such as Subrahmanyam and Greenfield [11] underline that healthy internet use can positively influence young people’s social skills, as they use online communication to strengthen the relationships they have already established with friends in the real world, as well as with their partners. In addition, they observed that young people use the network as an element from which they can obtain information about people who are relevant to their interests. These authors indicate that as the use of this tool has grown, so has contact with people who were strangers, producing benefits such as reduced anxiety when establishing social relationships. Similar ideas are shared by Solano, González, and López [12] who found that the appropriate use of the Internet in social relationships served to increase and improve the bond that already existed with acquaintances and family members in the real world. These authors emphasize that, in some cases, the use of the internet brings individuals who already have social ties closer together, and in this way, they can maintain contact. Virtual communication allows for an extension of the face-to-face space, as many of the young people confirmed that they used the Internet to arrange meetings with friends, with individuals with the best developed social skills being the biggest consumers of online communication [12]. Linked to this belief, Sabater, Martínez, and Campión [13] confirmed that the Internet is indeed a social tool that, used in a healthy way, allows for maintaining and strengthening social relationships that already existed in real life. However, they stress that they cannot replace the establishment of face-to-face social relations, since, in the virtual environment, aspects such as trust, the expression of feelings and appreciation are hindered, i.e., there is a lack of the elements that are necessary to establish affective links that are maintained over time. Therefore, the Internet provides a tool that complements previously established relationships and helps to ensure that distance and the passage of time do not affect them [13].

However, on the other hand, there are authors who argue that problematic internet use reduces the time spent on interpersonal relationships and can lead to social isolation [14,15]. Increased time spent on the Internet could lead to a decrease in face-to-face communication and thus a decrease in social interaction [14]. In addition, people who believe that they lack good social skills and have poor communication skills may think that interacting through the Internet can hide these shortcomings [6,7,8,15]. They may also make the decision to avoid interacting and communicating with others and focus exclusively on surfing the Internet alone, thus avoiding facing their difficulties [15].

In this vein, Caplan [16] proposed that a symptom of problematic internet use is a preference for online rather than face-to-face social interaction. According to this author, online interaction is perceived by people with lower social skills as more effective, safer, more comfortable and more trusting than face-to-face interpersonal relationships. There are several reasons why people with social problems may develop a strong preference for online social interaction. For example, computer-mediated communication via an online chat room implies greater control over self-presentation, greater anonymity, lower perceived social risk (i.e., if social relationships were to fail, there would be less personal cost), more intimate and intense self-disclosure and less social responsibility towards others compared to traditional face-to-face communication [16].

Adolescents and young people, who see the internet as an indispensable part of their daily lives, constitute the most important risk group in terms of problematic internet use [17,18]. During this stage, young people seek to establish links with their peers and to have different forms of autonomy and independence [18]. Therefore, the use of the internet can be an attractive tool as a means of socializing. With the transition from adolescence to adulthood, the amount of time spent on the Internet increases [19]. Young adults, especially college students, are at an increased risk of problematic internet use due to the ease of access, either via mobile phone or computer, on college campuses [20]. Moreover, it is a stage where the management of time spent on the Internet becomes the responsibility of the individual, and the family takes a back seat. Young university students enjoy freedom and a lack of parental control over what they say and do online, as most of them live away from their parents or in university dorms [19,20]. Similarly, internet use by university students is very varied and includes activities such as searching for academic information, communicating and interacting with friends through social networks, downloading and watching videos and films and playing online games [20]. For this reason, as adolescents and young adults have become the group that makes the most use of the Internet, research on problematic internet use has increased at this stage [21,22]. However, previous studies generally focus on all university students, without making distinctions between different study programs [19,20]. The number of studies that focus on university students who are to be future teachers, educators or social workers is very limited [23]. The study of this particular population can be very interesting, as these students will be the ones educating, training and developing programs to prevent and intervene in problematic internet use. As such, there is a need for studies focusing specifically on problematic internet use among students in faculties of education, with the aim of finding out which risk factors are predictors of problematic internet use. In this way, the curricula of faculties of education could include training on reducing problematic internet use and could train future educators on how they can encourage healthy internet use in themselves and their future students. Moreover, this analysis is particularly relevant given that one of the goals of the 2030 Agenda for Sustainable Development focuses on promoting affordable and universal access to the Internet for the least developed countries. Knowledge of the risk factors could facilitate the development of programs to prevent problematic internet use and the consequences of such use. Therefore, this research aims to analyze the extent to which social skills and age predict problematic internet use. Although there are some studies on this issue, there is still a need to clarify the existing doubts about the role of social skills in problematic internet use. In addition, some dimensions of social skills that have not been studied, such as empathy, are included in this research. Empathy could be a predictor of internet use because more empathetic people may consider the feelings, emotions and thoughts of the people around them.

Thus, they may feel bad when they use the Internet intensively as they feel that they are neglecting the people around them and making them feel bad.

## 2. Materials and Methods

### 2.1. Participants

The research consisted of a study population of 514 participants, 48 males and 466 females, aged between 17 and 52 years (Median age = 22.20 years, SD Age = 3.86). All participants were university students enrolled at a university in southeastern Spain, of which 6.4% were graduate students and 93.6% were undergraduate students. The undergraduate students were pursuing different university degrees, namely 7.8% were studying a degree in Social Work, 8.8% a degree in Social Education, 71.4% a degree in Early Childhood Education and 5.6% a degree in Primary Education. All postgraduate participants, i.e., 6.4%, were taking the master’s degree in Compulsory Secondary Education, Vocational Training and Language Teaching.

### 2.2. Instrument

For the assessment of problematic internet use, the Generalized Problematic Internet Use Scale, in its revised version (GPIUS2), was used [24,25]. It is a 15-item Likert-type scale with 6 response options ranging from 1 (strongly disagree) to 6 (strongly agree). It consists of 4 subscales: preference for online social interaction, mood regulation through the Internet, negative consequences and deficient self-regulation. Preference for social interaction is composed of 3 items and consists of the belief that, compared to face-to-face interactions, relationships conducted over the Internet are more reliable, more pleasant, more effective and less disturbing (e.g., “I prefer to communicate with people over the Internet rather than face-to-face”). Mood regulation through the Internet, also consisting of 3 items, refers to using the Internet to minimize emotional distress or feelings of loneliness (3 items, e.g., “I used the Internet to feel better when I felt angry”). The negative consequences scale assesses the extent to which a person experiences problems with their social, work, personal or academic environment as a result of inappropriate internet use (3 items, e.g., “my Internet use has created problems in my life”). Finally, deficient self-regulation is defined as a structure that includes compulsive internet use and cognitive preoccupation. Compulsive internet use consists of 3 items that refer to the inability to regulate or master internet access (e.g., “When I am not on the Internet, it is difficult to resist the urge to go online”). Finally, cognitive preoccupation includes 3 items assessing the inability to control obsessive thoughts related to internet use (e.g., “I obsessively think about going online when I am not”). Cronbach’s alphas obtained for this research are α = 0.84 for preference for online social interaction, α = 0.79 for mood regulation through the Internet, α = 0.74 for negative consequences and α = 0.86 deficient self-regulation (α = 0.82 for compulsive internet use and α = 0.69 for cognitive preoccupation).

The Inventory of Social Skills (IHS), adapted to Spanish, was used to assess social skills [26,27]. The questionnaire is based on a Likert scale with 5 response options ranging from rarely or never to almost always or always. It is made up of 5 scales: conversation and social ease, which consists of 7 items and assesses the ability to start, maintain and end verbal interactions with different people and related to the ability to handle oneself adequately in conversations without suffering excessive social anxiety (e.g., “even with acquaintances from school or work, I find it difficult to integrate myself into a conversation”); empathic and positive feeling skills, consisting of 9 items that refer to a person’s ability to convey affection, praise, compliments, esteem, feelings and personal opinions when the positive behavior of others demonstrates that they have a legitimate reason. It also includes the ability to put oneself in the position of others and to safeguard their rights. This capacity makes subjects good deliverers of social enhancement (e.g., “In a group situation, when someone is treated unfairly, I react in their defense”); self-exposure to strangers and new situations, which includes 6 items related to approaching strangers who are important or pleasant to the individual (e.g., “When I feel like meeting someone to whom I have not been introduced, I introduce myself”); coping with risk, consisting of 6 items assessing the subject’s ability to confront or avoid an opponent’s unacceptable behavior or comments and the ability to achieve more appropriate behavior in the future. Conceptually, this factor is related to the term assertive opposition, whereby people can effectively communicate their position (e.g., “In a conversation, if a person interrupts me, I ask them to wait until I finish what I was saying”); academic and work social skills which includes 4 items assessing interpersonal competencies required for a successful professional and academic occupation, such as the ability to ask questions and present publicly in formal contexts (e.g., “I make presentations (e.g., at a conference, in the classroom or at work when asked to do so”). Cronbach’s alphas for this research are α = 0.73 for conversation and social ease, α = 0.76 for empathic and positive feeling skills, α = 0.79 for self-exposure to strangers and new situations, α = 0.71 for coping with risk and α = 0.77 for academic and work social skills.

### 2.3. Procedure

In order to carry out the research, first of all, collaboration was requested from different lecturers at the University of Granada (Spain) in charge of teaching in different bachelor’s and master’s degrees.

Those teachers who decided to collaborate facilitated the beginning of their class time to carry out the research. Initially, a link was sent to the students explaining the title of the study, indicating the importance of answering the questionnaires honestly, given that they were anonymous questionnaires, and offering them the possibility of revoking their consent at any time and without having to give explanations, given that their participation was voluntary. In addition, this initial information ensured data protection and students were asked to tick a box if they agreed with the information read and gave their consent to participate in the study. Once the informed consent box had been ticked, the students who decided to participate went on to complete the questionnaires. Once the questionnaires were completed, the data were transferred to a database where they were analyzed anonymously. Finally, the research report was written up.

### 2.4. Design and Statistical Analysis

For the present research, a prospective Ex Post-Facto design was used as the objective was to analyze the extent to which the dependent variable, i.e., problematic internet use, was explained by the independent variables (age and social skills variables).

Descriptive statistics (means, standard deviations and minimum and maximum scores) were then calculated for each of the variables under study.

Subsequently, multivariate multiple linear regression models were used to determine the influence of all the social skills variables considered and of age on each of the variables related to problematic internet use. Social skills and age were included in the model as independent or predictor variables and each of the problematic uses of the Internet (preference for online social interaction; mood regulation through the Internet; negative consequences; deficient self-regulation) were included as dependent variables. The degree of statistical significance for all hypothesis-contrast tests was set at *p* < 0.05.

The program used for the analyses was Statistical Package for the Social Sciences (SPSS) version 26 for Mac.

## 3. Results

Table 1 shows the mean scores and standard deviations of the participants for each of the social skills and Internet use variables.

On the other hand, in order to find out whether any of the social skills variables considered predicted the scores of each of the problematic internet use variables, multiple linear regression was carried out. In this sense, the results of the reduced multiple linear regression models for the variable preference for online social interaction are presented in Table 2. Including all the independent variables considered in the model, in order to know the influence on the dependent variable, it is observed that there are two social skills variables that obtain a significant negative partial correlation (conversation and social ease and empathic and positive feeling skills). Thus, lower scores on these two skills predict higher preference for online social interaction. The model as a whole, although significant, explained only 6% of the variance.

Table 3 shows the results found for the variable mood regulation through the Internet. In this case, two social skills variables were also found to be significant predictors of mood regulation through the Internet: conversation and social ease and coping with risk. However, the conversation and social ease variable is partially negatively correlated, while coping with risk is positively correlated. In this sense, lower scores on conversation and social ease and higher scores on coping with risk predict higher levels of mood regulation through the Internet. On the other hand, age also showed a negative partial correlation, being a predictor of mood regulation through the Internet. Therefore, younger age predicts a higher mood regulation through the Internet score. The prediction model with all variables included was found to be significant, explaining 3% of the variance.

As shown in Table 4, two variables included in the prediction model were significant for the negative consequences of internet use: on the one hand, conversation and social ease and, on the other hand, empathic and positive feeling skills. The likelihood of high scores on negative consequences is higher as participants lower their scores on conversation and social ease and on empathy and expressing positive feelings skills. The other social skills variables were not predictive of negative consequences, as was the case with age. The model predicts only 6% of the variance.

Finally, Table 5 shows the results obtained for the deficient self-regulation variable. In this case, conversation and social ease and empathic and positive feeling skills were found to be significant predictors, with negative partial correlations in both cases. Thus, lower scores on both skills predict higher scores on deficient self-regulation. Similarly, age is a significant predictor of deficient regulation, with the likelihood of having high poor regulation increasing as the age of participants decreases. The overall model, considering all the variables that had been included, explains 3% of the variance.

## 4. Discussion

Technological developments in recent years have meant that people are increasingly using the Internet in their daily lives. Although the increase in internet use makes people’s lives easier in many ways, excessive use of the Internet can lead to problems. To promote the prevention and early intervention of problematic internet use, it is imperative to identify the risk factors and underlying mechanisms of problematic internet use. For this reason, this research aimed to test whether age or social skills are predictors of problematic internet use.

First, the extent to which age predicted problematic internet use was analyzed. In this regard, age was found to be a predictor variable of mood regulation through the Internet and deficient self-regulation. Younger students were more likely to use the Internet to minimize loneliness or feelings of emotional distress and were more likely to develop problems controlling obsessive thoughts about internet use and to engage in compulsive internet use compared to older students. Similar results were found by Sechi et al. [18] who conducted research with 362 university students and found that age negatively affects problematic internet use, with younger people being more likely to develop addictive internet behavior than older people. Similarly, Akhter et al. [20] conducted research with 432 university students and found that young university students had more problematic internet use compared to the senior university students in the study.

Furthermore, Ak, Koruklu, and Yılmaz [28] indicated that, compared to other addictions, problematic internet use starts at an early age. Adolescents and young adults spend many hours on the Internet, chatting with peers, playing online games or checking their social networks, and these factors increase the likelihood of developing problematic internet use among university students [22].

However, in the present research, age did not predict all variables of problematic internet use. No age prediction was found for the preference for online social interaction and negative consequences. Thus, it appears that, while age does play a predictive role in the use of the Internet to reduce emotional distress, in the control of obsessive thoughts and compulsive internet use, it does not appear to determine preference for online rather than face-to-face interactions or the impairment of social, personal and academic relationships as a result of problematic internet use.

Finally, this study aimed to find out to what extent social skills predicted problematic internet use. The conversation and social ease variable predicted all variables of problematic internet use (preference for online social interaction, mood regulation through the Internet, negative consequences and deficient self-regulation). Thus, people who found it easier to initiate, maintain and end conversations with others without experiencing social anxiety were less likely to prefer online social interaction and to use the Internet to regulate their mood. In addition, these students were less likely to have obsessive thoughts related to internet use, were more likely to regulate their internet access and had fewer problems in their social, work, personal or academic environment as a result of problematic internet use.

On the other hand, empathic and positive feeling skills predicted preference for online social interaction, negative consequences and deficient self-regulation. Students who were more empathetic and more likely to express positive feelings were less likely to prefer online interaction, had fewer social, personal, work or academic problems as a result of inappropriate internet use and were more likely to control their thoughts and time spent online. Although the role of empathy in problematic internet use has not been of interest to researchers, this research shows that it may play an important role in predicting problematic internet use. Therefore, it seems advisable to further investigate the role that empathy plays in the problematic internet use and the effect of this use on adolescents and young adults.

In relation to the variable self-exposure to strangers and new situations, it did not predict any variables of problematic internet use. Therefore, students’ ability to approach strangers with the aim of getting to know them or initiating a conversation does not seem to be a determinant of problematic internet use.

On the other hand, the coping with risk variable predicted mood regulation through the Internet. In this sense, more assertive students, able to reject unfair comments or behavior, were more likely to use the Internet to minimize emotional distress or feelings of loneliness.

Finally, the academic and work social skills variable did not predict any problematic internet use variables. Therefore, it seems that the interpersonal skills needed to be successful in a professional and academic occupation do not play a relevant role in problematic internet use.

Although the role of social skills in problematic internet use has been studied before, the results are inconclusive. On the one hand, Valkenburg and Peter [29] conducted a study involving 1158 adolescents and found that the use of the Internet as a communication tool favored both social skills and self-concept among students. Similar results were found by Solano et al. [12], who wanted to observe the similarities and differences between virtual and face-to-face communication of adolescents, as well as to study whether the introduction of the Internet had affected the type of interaction. They used a sample of 3103 individuals aged between 14 and 16 years. The results showed that those young people who most frequently used the Internet and social networks as a means of communication corresponded to individuals with the most developed social skills.

Similarly, there are multiple studies that argue that adequate social skills development may be a protective factor for problematic internet use. For example, the study by Delgado et al. [6] involving 1405 university students found that students with better social skills were less likely to be addicted to social networks. Similarly, Odaci et al. conducted a study involving 480 students and found that inhibitory interpersonal relationships, automatic thoughts and problem-solving skills are predictors of problematic internet use [15].

Similar conclusions were reached by Terroso and Argimon [8]. These authors conducted a study with 482 students on the link between internet dependence and the development of social skills. The results showed that adolescents with difficulties in social skills were the same adolescents who reported higher levels of technological addiction. Therefore, they concluded that despite the benefits of the Internet in people’s lives, problematic Internet use by people with poor social skills can lead to the development of addiction.

Finally, Estrada-Araoz et al. [7] carried out a study on social skills and people’s predisposition to suffer from symptoms of virtual addiction, in which they included a sample of 102 students in the fifth year of secondary education in an area of Peru. After analyzing the data, they concluded that there is a predisposition to compulsive and problematic Internet use the less developed the social skills are.

In the present research, although all of the social skills variables did not predict all of the problematic internet use variables, it was found that there are several social skills variables that predict problematic internet use, with the conversation and social ease variable predicting the largest number of problematic internet use variables. These results suggest the need for prevention and intervention programs to reduce problematic internet use, and it is important that these programs work on social skills to help young people improve interpersonal management skills and reduce the likelihood of problematic internet use.

Despite the results found, this research has some limitations that are important to bear in mind. Firstly, this is a cross-sectional study and therefore does not allow us to establish whether social skills and age predict problematic internet use over time. It would be interesting to conduct longitudinal studies to see whether this prediction holds or changes over time. On the other hand, the study recruited only university students enrolled at a university in Spain, so these results are difficult to extrapolate. Furthermore, as discussed in the introduction, the concept of social skills varies according to the cultural context of the individual. It would therefore be interesting to replicate the research in other countries. Another limitation of the study is related to gender, as the sample consisted mainly of women, which could bias the results found. Similarly, in this research only two variables were considered as predictors of problematic internet use (age and social skills). Future lines of research could consider the role of other personal and contextual variables, such as family type, gender or self-regulatory capacity. In addition, problematic internet use is analyzed in a generic way. It is not specified what specifically it is used for (e.g., playing video games, chatting, online shopping, etc.), the media used (e.g., mobile phone, computer, etc.) and the time students spend online. It would be interesting for future studies to qualify these aspects.

## 5. Conclusions

The following conclusions can be drawn from the results obtained. Firstly, both age and social skills influenced the prediction of problematic internet use. While it is true that social skills did not predict all variables of problematic internet use, some social skills variables (conversation and social ease, empathic and positive feeling skills, risk coping) predicted problematic internet use. Similarly, age appears to be a predictor of preference for online social interaction and deficient self-regulation, which encompasses compulsive internet use and cognitive preoccupation. However, it does not seem to play a relevant role in the consequences negatives of problematic internet use and in mood regulation through the Internet.

## Figures and Tables

**Table 1:** Descriptive data on social skills (Independent variable) and internet use (Dependent variable).

Social Skills	M (SD)	Min.	Max.
Conversation	25.15 (4.40)	10.00	35.00
Empathic	35.30 (5.54)	15.00	45.00
Self-exposure to strangers	18.29 (4.58)	6.00	30.00
Coping with risk	16.65 (3.79)	6.00	25.00
Academic and work social skills	12.31 (3.32)	4.00	20.00
**Internet Use**			
Preference for online social interaction	5.11 (2.84)	3.00	18.00
Mood regulation through the Internet	10.03 (3.98)	3.00	18.00
Negative consequences	5.23 (2.36)	2.00	15.00
Deficient self-regulation	15.56 (6.24)	6.00	34.00

M = Media. SD = Standard Deviation. Min. = Minimum. Max. = Maximum. Conversation = conversation and social ease. Empathic = empathic and positive feeling skills. Self-exposure to strangers = self-exposure to strangers and new situations.

**Table 2:** Summary of the multiple linear regression model for the preference for online social interaction variable in line with the predictor variables included in the analysis.

						95% C.I	
Variable	Predictor	β	E.S	t	*p*	Lower	Upper
Preference for online social interaction	Age	0.008	0.032	0.19	0.846	−0.06	0.07
	Conversation	−0.174	0.032	−3.51	−0.000	−0.17	−0.05
	Empathic	−0.126	0.025	−2.58	0.010	−0.11	−0.02
	Self-exposure to strangers	−0.062	0.033	−1.15	0.253	−0.10	0.03
	Coping with risk	−0.020	0.036	−0.41	0.680	−0.09	0.05
	Academic and work social skills	0.013	0.044	0.25	0.804	−0.07	0.09

β = Standardized regression coefficient. SE = Standard error. Conversation = conversation and social ease. Empathic = empathic and positive feeling skills. Self-exposure to strangers = self-exposure to strangers and new situations. Coefficient of determination (adjusted R^2^) = 0.062, *F* = 6.62, *p* = 0.001.

**Table 3:** Summary of the multiple linear regression model for mood regulation through the Internet variable in line with the predictor variables included in the analysis.

						95% C.I	
Variable	Predictor	β	E.S	t	*p*	Lower	Upper
Mood regulation through the Internet	Age	−0.119	0.045	−2.72	0.007	−0.21	−0.03
	Conversation	−0.122	0.046	−2.42	0.016	−0.20	−0.02
	Empathic	−0.068	0.036	−1.37	0.173	−0.12	0.02
	Self−exposure to strangers	0.024	0.047	0.44	0.658	−0.07	0.11
	Coping with risk	0.147	0.051	3.04	0.002	0.05	0.25
	Academic and work social skills	0.026	0.062	0.49	0.622	−0.09	0.15

β = Standardized regression coefficient. SE = Standard error. Conversation = conversation and social ease. Empathic = empathic and positive feeling skills. Self-exposure to strangers = self-exposure to strangers and new situations. Coefficient of determination (adjusted R^2^) = 0.029, *F* = 3.57, *p* = 0.002.

**Table 4:** Summary of the multiple linear regression model for negative consequences variable in line with the predictor variables included in the analysis.

						95% C.I	
Variable	Predictor	β	E.S	t	*p*	Lower	Upper
Negative consequences	Age	−0.084	0.026	−1.94	0.053	−0.10	0.01
	Conversation	−0.171	0.027	−3.45	0.001	−0.14	−0.04
	Empathic	−0.210	0.021	−4.30	0.000	−0.13	−0.05
	Self-exposure to strangers	0.067	0.028	1.25	0.211	−0.02	0.09
	Coping with risk	0.017	0.030	0.35	0.728	−0.05	0.07
	Academic and work social skills	0.024	0.037	0.46	0.646	−0.05	0.09

β = Standardized regression coefficient. SE = Standard error. Conversation = conversation and social ease. Empathic = empathic and positive feeling skills. Self-exposure to strangers = self-exposure to strangers and new situations. Coefficient of determination (adjusted R^2^) = 0.057, *F* = 6.18, *p* = 0.001.

**Table 5:** Summary of the multiple linear regression model for deficient self-regulation variable in line with the predictor variables included in the analysis.

						95% C.I	
Variable	Predictor	β	E.S	t	*p*	Lower	Upper
Deficient self-regulation	Age	−0.120	0.071	−2.75	0.006	−0.33	−0.05
	Conversation	−0.117	0.071	−2.33	0.020	−0.31	−0.03
	Empathic	−0.126	0.056	−2.54	0.011	−0.25	−0.03
	Self-exposure to strangers	0.059	0.074	1.07	0.283	−0.07	0.23
	Coping with risk	0.033	0.080	0.67	0.501	−0.10	0.21
	Academic and work social skills	−0.040	0.098	−0.76	0.447	−0.27	0.12

β = Standardized regression coefficient. SE = Standard error. Conversation = conversation and social ease. Empathic = empathic and positive feeling skills. Self-exposure to strangers = self-exposure to strangers and new situations. Coefficient of determination (adjusted R^2^) = 0.033, *F* = 3.92, *p* = 0.001.

## Data Availability

Data available on request due to ethical restrictions.
